# The association between nutritional status measured by body mass index and outcomes in the pediatric intensive care unit

**DOI:** 10.3389/fped.2024.1421155

**Published:** 2024-09-17

**Authors:** Zahra Pournasiri, Mahsa Bakhtiary, Ali Nikparast, Seyedeh Masumeh Hashemi, Seyyedeh Narjes Ahmadizadeh, Azita Behzad, Golaleh Asghari

**Affiliations:** ^1^ Pediatric Nehrology Research Center, Research Institute for Children’s Health, Shahid Beheshti University of Medical Sciences, Tehran, Iran; ^2^ Pediatric Nephrology Research Center, Research Institute for Children’s Health, Mofid Children’s Hospital, Shahid Beheshti University of Medical Science, Tehran, Iran; ^3^ Pediatric Gastroenterology and Hepatology Research Center, Pediatrics Centre of Excellence, Children’s Medical Center, Tehran University of Medical Sciences, Tehran, Iran; ^4^ Department of Clinical Nutrition & Dietetics, Faculty of Nutrition Science and Food Technology, Shahid Beheshti University of Medical Sciences, Tehran, Iran; ^5^ Pediatric Intensive Care Departmant, Mofid Children Hospital, Shahid Beheshti University of Medical Sciences, Tehran, Iran

**Keywords:** pediatric intensive care unit (PICU), nutritional status, mortality, prolonged PICU stay, children

## Abstract

**Aim/Introduction:**

The relationship between nutritional status upon admission to a pediatric intensive care unit (PICU) and clinical outcomes remains unclear. We examined the relationship between nutrition status, as indicated by body mass index-for-age (BMI-for-age), and clinical outcomes in the PICU.

**Method:**

In this retrospective study at a tertiary care center, records of 1,015 critically ill children and adolescents aged one month to 18 years old with available anthropometric parameters were included. The nutritional status upon admission was determined by calculating the BMI-for-age *z*-score using the WHO growth charts as the reference. The participants were categorized as underweight (BMI-for-age *z*-score < −2), normal weight (−2 ≤ BMI-for-age *z*-score ≤ +1), and overweight/obese (BMI-for-age *z*-score > +1). Multi-variate odds ratios (OR) with 95% confidence intervals (CI) were used to investigate the association between malnutrition (being underweight and overweight/obese) and odds of Prolonged PICU stay (≥7 days) and PICU mortality after controlling for descriptive characteristics, Glasgow Coma Scale score status, fluctuations in serum sodium, and acute kidney injury confounders.

**Results:**

The proportions of patients in underweight, normal weight, and overweight/obese categories were 34.2%, 45.8%, and 20%, respectively. During the study period, 21.5% of patients had prolonged PICU stay, and 5.6% of patients in PICU died. Compared to normal-weight patients, underweight patients had higher odds of prolonged PICU stay (OR: 1.52; 95% CI: 1.05–2.22) and PICU mortality (OR: 2.12; 95% CI: 1.22–4.01). Age- and gender-stratified full-adjusted analysis showed that the increased odds of prolonged PICU stay remained significant among underweight boys and underweight individuals aged 5–19 years old. Furthermore, the increased odds of PICU mortality remained significant among underweight individuals aged 2–5 years old. However, being overweight or obese during PICU admission did not demonstrate a significant association with our outcomes in the total sample or subgroup analysis.

**Conclusion:**

Our findings showed that PICU patients who were underweight had higher odds of prolonged PICU stay and PICU mortality than their normal-weight counterparts. This underscores the importance of closely monitoring underweight patients in the PICU upon admission in order to improve clinical outcomes.

## Introduction

Hospitalized patients frequently experience nutritional disorders, with malnutrition being the most prevalent (1, 2). Malnutrition, as defined by the World Health Organization (WHO), encompasses disparities in an individual's energy and nutrient consumption, including being underweight and overweight or obese (3). Malnutrition is a major concern in children admitted to pediatric intensive care units (PICUs), with reported rates reaching up to 53% (4, 5). Alongside the rising global prevalence of pediatric obesity (6), which is suggested to be associated with poorer outcomes in PICUs, there is more concern about suboptimal nutritional status (5, 7). In particular, being underweight is linked to a higher risk of prolonged PICU stay and PICU mortality (8, 9). On the other hand, the relationship between obesity and the risk of morbidity and mortality among critically ill children and adolescents remains unclear (10–12). In 2019, a meta-analysis showed that critically ill children and adolescents who were obese had an increased risk of mortality and a higher hospital length of stay in comparison to their non-obese counterparts (13). However, a systematic review and meta-analysis conducted by Toh S et al. failed to find any association between PICU admission BMI status and outcomes in critically ill children (9). In order to understand the ongoing debates, it seems that previous studies that evaluated the association between nutritional status and clinical outcomes in the PICU only adjusted for limited confounders such as participant descriptive characteristics (including age, sex, ethnicity, etc.) and did not take into consideration other significant risk factors like Glasgow Coma Scale (GCS) score, fluctuations in serum sodium and acute kidney injury (AKI) upon the initial days of admission to the PICU which is independent predictors of PICU admission clinical outcomes (14–17).

In order to address this gap in the existing body of research, our study aimed to elucidate the association between BMI at PICU admission, known as a widely accepted measure for assessing nutrition status, and the risk of prolonged PICU stay (≥7 days) and PICU mortality.

## Method

The present retrospective study was performed using medical records of children and adolescents aged between 1 month and 18 years old who were admitted to Mofid Children Hospital PICU, Tehran, Iran, from April 2019 through May 2022. The current study protocol was approved by the institute ethics committee of Shahid Beheshti University of Medical Sciences. Since this study was conducted retrospectively and involved collecting information from existing case records, obtaining written informed permission was not required. The exclusion criteria were as follows: Patients who were admitted to the PICU for less than 24 h, as well as those for whom demographic, anthropometric, and related biochemical measurements were not documented. Demographic information was collected upon the patient's admission and documented in the unit's database, which is standard procedure for admitting and discharging patients. The unit's research team regularly conducted reviews of this database. The weight of infants under two years old who lay still was measured using a standard protocol. As well, the weight of children and adolescents over two years old who were capable of standing was measured with the assistance of parents or nurses. Otherwise, the most recent weight recorded or measured before the PICU admission or determined at the time of hospital admission was utilized. The height was measured when the individual was lying down in a position where their head was neutral and their legs were fully extended at the knee and ankle. A measuring tape that does not stretch was used for the measurement.

The nutritional status upon admission was determined by calculating the BMI-for-age *z*-score values using the latest internationally recognized growth chart standards and references (18, 19). The *z*-scores were computed utilizing the World Health Organization AnthroPlus program (20). Participants were classified into different groups based on their BMI-for-age *z*-scores. Those with a BMI-for-age *z*-score value less than −2 were classified as underweight; those with a BMI-for-age *z*-score value between −2 and +1 were classified as normal weight; those with a BMI-for-age *z*-score value between +1 and +2 were classified as overweight; and those with a BMI for age *z*-score value greater than +2 were classified as obese (18, 21). As there were not enough participants in the obese category, the overweight and obese groups were merged into one category, referred to as the overweight/obesity category. The GCS score was assessed by the pediatric resident at the time of admission (22). An individual's GCS score may vary between 3 (indicating complete unresponsiveness) and 15 (indicating full responsiveness). The GCS score was categorized as severe (GCS < 8), moderate (8 < GCS < 13), and mild (GCS > 13) (23, 24). AKI was diagnosed using the Acute Kidney Injury Network (AKIN) criteria, which involves identifying an absolute increase in serum creatinine (SCr) level of at least 0.3 mg/dl or a 50% (1.5-fold) increase in SCr from the baseline within 48 h of a bilateral kidney insult (25). The fluctuations in serum sodium levels were calculated as the difference between the maximum and minimum values of sodium concentration up to 7 days following PICU admission. The fluctuations in serum sodium levels were divided into quartiles, with Quartile 1 denoting the lowest serum sodium fluctuation and Quartile 4 denoting the highest (indicating a worse status). Blood sampling was done on the days of admission to assess the serum concentration of sodium and creatinine. Sodium was measured using an ion selective electrode (ISE) assay by Caretium XI-921 (made in China). Creatinine was measured using Jaffe. Colorimetric-Kinetic assay by BioLis 24i (made in Japan).

The independent variables in this study were underweight and overweight/obese (with normal weight as the reference group). The binary primary outcomes of the current study were as follows: (1) Prolonged PICU stay (≥7 days) and (2) PICU mortality.

## Statistical analysis

Demographic and clinical characteristics of individuals across nutrition status categories were described using standard descriptive statistics analysis. The normality of distribution was assessed using the Kolmogorov-Smirnov goodness-of-fit analysis. Continuous variables with a normal distribution are typically presented as means (± standard deviation, SD) and compared between different nutritional status categories applying analysis of variance (ANOVA). Variables that exhibit non-normal distribution are presented as medians, accompanied by interquartile ranges (IQR), and are compared between nutritional status categories using the Kruskal-Wallis test. Categorical variables are expressed as proportions, accompanied by percentages, and are compared between nutritional status categories using either chi-square or Fisher's exact tests, depending on the circumstances. The association between nutritional status and odds of prolonged PICU stay (≥7 days), as well as PICU mortality, were evaluated by calculating the multiple-adjusted odds ratios (ORs) using logistic regression analysis. The ORs and 95% confidence intervals (CIs) were evaluated for nutritional status as categorical variables for the total sample and age and sex-stratified subgroups. Accordingly, three models were constructed: (1) crude model; (2) model 1, adjusted for age and gender; and (3) model 2, additionally adjusted for GCS score status, fluctuations in serum sodium, and AKI. The statistical analyses were performed using SPSS version 20 (SPSS, Chicago, IL, USA), with a significance level of <0.05 for a two-tailed *P*-value.

## Results

A total of 1,126 patients were recorded during the study period. We excluded 102 patients with a length of stay in PICU less than 24 h and nine patients with insufficient data to calculate a BMI-for-age *z*-score. Finally, 1,015 patients (including 441 girls and 574 boys) were included in our analysis. Based on the BMI-for-age *z*-score categories, the proportions of patients with underweight, normal weight, and overweight/obese categories were 34.2% (*n* = 347), 45.8% (*n* = 465), and 20% (*n* = 203), respectively. According to the categories of admission type, the proportion of patients with elective surgery, emergency surgery, and medical were 18.8% (*n* = 191), 6% (*n* = 61), and 75.2% (*n* = 763), respectively. The distribution of GCS score status categories was as follows: 65.8% classified as mild, 12.6% as moderate, and 21.6% as severe. The mean ± SD of serum sodium and creatinine levels were 136.9 ± 5.1 mmol/L and 0.66 ± 0.47 mg/dl, respectively. During the study period, 60 (5.9%) participants experienced AKI. 561 (55.3%) participants had the lowest fluctuations in serum sodium levels, and 186 (18.3%) had the highest ones. The mean ± SD of PICU length of stay (days) was 5.9 ± 17.1. Based on the BMI-for-age *z*-score categories, PICU length of stay (days) in underweight, normal weight, and overweight/obese individuals were 7.0 ± 19.7, 5.3 ± 17.8, and 5.5 ± 9.0, respectively. During the study period, 218 patients with prolonged PICU stay (≥7 days) (21.5%) and 57 PICU mortality (5.6%) were recorded.

Table 1 represents the characteristics of patients by nutritional status. Underweight patients tended to be younger and had a higher distribution of severe GCS score status compared to those with normal weight or overweight-obese status. No significant differences between nutritional status categories were seen regarding gender, admission type, diagnosis categories, baseline serum sodium and creatinine level, AKI, fluctuations in serum sodium levels, PICU length of stay, and prolonged PICU stay.

**Table 1:** The characteristics of participants across nutrition status measured by body mass index.

	Total sample (*N* = 1,015)	Underweight (*Z*-score < −2) (*N* = 347)	Normal weight (−2 ≥ *Z*-score ≤ +1) (*N* = 465)	Overweight and obese (*Z*-score > +1) (*N* = 203)	*P*-value*
Boys (%)	574 (56.6)	206 (35.9)	260 (45.3)	108 (18.8)	0.34
Age (month)	54.3 ± 54.7	49.9 ± 55.2	53.8 ± 54.1	63 ± 54.4	0.02
Height (cm)	97.6 ± 32.4	95.7 ± 31.1	96.8 ± 31.9	102.6 ± 36.3	0.03
Weight (kg)	17.2 ± 14.8	11.9 ± 8.6	16.5 ± 12.1	27.2 ± 21.3	<0.001
Body mass index (kg/m^2^)	15.8 ± 7.3	11.7 ± 1.9	15.6 ± 1.6	23.0 ± 12.4	<0.001
Admission type					
Elective surgery (%)	191 (18.8)	71 (37.2)	93 (48.7)	27 (14.1)	0.22
Emergency surgery (%)	61 (6)	21 (34.4)	25 (41.0)	15 (24.6)	
Medical (%)	763 (75.2)	255 (33.4)	347 (45.5)	161 (21.1)	
Diagnosis category					
Cardiorespiratory (%)	129 (12.7)	47 (36.4)	56 (43.4)	26 (20.2)	0.21
Neuromuscular (%)	256 (25.2)	68 (26.5)	122 (47.6)	66 (25.9)	
Infectious disease (%)	77 (7.6)	26 (33.7)	36 (46.7)	15 (26.8)	
Hematology/oncology (%)	68 (6.7)	22 (32.3)	33 (48.5)	13 (19.2)	
Gastrointestinal (%)	129 (12.7)	52 (40.3)	56 (43.4)	21 (16.3)	
Renal, endocrine (%)	104 (10.2)	40 (38.4)	44 (42.3)	20 (19.3)	
Surgical (%)	191 (18.8)	71 (31.7)	93 (48.7)	27 (19.6)	
Emergency (%)	61 (6)	21 (34.4)	25 (41.0)	15 (24.6)	
Glasgow Coma Scale score status					
Mild (%)	668 (65.8)	204 (30.5)	313 (46.8)	151 (22.7)	<0.001
Moderate (%)	128 (12.6)	40 (31.2)	63 (49.2)	25 (19.6)	
Severe (%)	219 (21.6)	103 (47.0)	89 (40.6)	27 (12.4)	
Serum sodium level (mmol/L)	136.9 ± 5.1	136.6 ± 5.3	136.6 ± 4.9	137.1 ± 4.4	0.49
Serum creatinine level (mg/dl)	0.66 ± 0.47	0.64 ± 0.03	0.31 ± 0.14	0.32 ± 0.02	0.35
Acute kidney injury (%)	60 (5.9)	22 (36.6)	27 (45)	11 (18.4)	0.87
Fluctuations in Serum Sodium levels					
Q1	561 (55.3)	183 (32.6)	263 (46.9)	115 (20.5)	0.71
Q2	174 (17.1)	66 (37.9)	78 (44.8)	30 (17.3)	
Q3	94 (9.3)	35 (37.2)	37 (39.3)	22 (23.6)	
Q4	186 (18.3)	59 (31.7)	88 (47.3)	39 (21)	
PICU length of stay (days)	5.9 ± 17.1	7.0 ± 19.7	5.3 ± 17.8	5.5 ± 9.0	0.37
Prolonged PICU stay (%)	218 (21.5)	87 (39.9)	88 (40.4)	43 (19.7)	0.11
PICU mortality (%)	57 (5.6)	31 (54.4)	18 (31.6)	8 (14)	<0.01

Data represented as mean ± standard deviation, or median (interquartile) for continuous variables and number and percent for categorical variables.

* Chi-square or Fisher's exact, and analysis of variance or Kruskal-Wallis tests were used to test the categorical and continuous variables across nutritional status categories.

Supplementary Table S1 represents the characteristics of participants across gender. Briefly, the boys, compared to girls, had a higher prevalence of moderate and severe GCS score status (moderate GCS status: 59.4% of boys vs. 40.6% of girls; severe GCS status: 62.6% of boys vs. 34.7% of girls and higher PICU mortality (boys: 78.9% VS girls: 21.1%). No significant differences between boys and girls were seen regarding age, height, weight, BMI, serum sodium and creatinine level, admission type, diagnosis category, AKI, fluctuations in serum sodium levels, PICU length of stay, and prolonged PICU stay.

Supplementary Table S2 shows the characteristics of participants across age groups. Participants aged 0–2 years old compared to those aged 2–5 or 5–19 had higher elective, emergency, and medical surgery distribution. No significant differences between age groups were seen regarding the patient's GCS score status category, serum sodium levels, PICU length of stay, prolonged PICU stay, and PICU mortality.

Table 2 reported the OR and 95% CI of nutritional status for odds of prolonged PICU stay (≥7 days) in total, as well as the sex and age-stratified subgroup analysis. In the total sample, underweight patients had significantly higher odds of prolonged PICU stay (≥7 days) in the crude model (OR: 1.43; 95% CI: 1.02–2.01), adjusted model 1 (OR: 1.42; 95% CI: 1.02–1.99), and adjusted model 2 (OR: 1.52; 95% CI: 1.05–2.22). According to gender-stratified analysis, the increased odds of prolonged PICU stay (≥7 days) for patients with underweight remained significant in boys in crude and adjusted models (adjusted OR and 95% CI were as follows: adjusted model 1: OR: 1.69; 95% CI: 1.10–2.60, and adjusted model 2: OR: 1.84; 95% CI: 1.13–2.98). As well, age-stratified analysis showed that underweight patients aged 5–19 years old had higher odds of prolonged PICU stay (≥7 days) in the crude model (OR: 2.58; 95% CI: 1.43–4.67), adjusted model 1 (OR:2.46; 95% CI: 1.35–4.47), and adjusted model 2 (OR: 2.48; 95% CI: 1.30–4.76). However, the increased odds of prolonged PICU stay (≥7 days) for patients with underweight aged 0–2 or 2–5 years lost its statistically significant level. Furthermore, there was no significant association between being overweight/obese and prolonged PICU stay (≥7 days), neither in crude or adjusted models in total nor in age- or gender-stratified analysis.

**Table 2:** The association between nutritional status measured by body mass *z*-score and risk of prolonged PICU stay (>7 days).

	Underweight (*Z*-score < −2)		Normal weight (−2 ≥ *Z*-score ≤ +1)	Overweight and obese (*Z*-score > +1)	
	OR (95% CI)	*P*-value		OR (95% CI)	*P*-value
Total sample					
Crude model	**1.43 (1.02–2.01)**	**0.03**	Ref	1.15 (0.76–1.73)	0.49
Model 1	**1.42 (1.02–1.99)**	**0.04**	Ref	1.21 (0.80–1.84)	0.35
Model 2	**1.52 (1.05–2.22)**	**0.03**	Ref	1.19 (0.75–1.91)	0.45
Gender stratification					
Boys					
Crude model	**1.68 (1.10–2.58)**	**0.02**	Ref	1.05 (0.60–1.83)	0.87
Model 1	**1.69 (1.10–2.60)**	**0.02**	Ref	1.12 (0.63–1.98)	0.69
Model 2	**1.84 (1.13–2.98)**	**0.01**	Ref	1.14 (0.58–2.23)	0.71
Girls					
Crude model	1.07 (0.60–1.86)	0.79	Ref	1.29 (0.71–2.35)	0.41
Model 1	1.07 (0.62–1.86)	0.80	Ref	1.30 (0.71–2.39)	0.39
Model 2	1.09 (0.59–1.99)	0.78	Ref	1.19 (0.61–2.32)	0.59
Age stratification					
0–2 years					
Crude model	1.09 (0.67–1.77)	0.74	Ref	1.72 (0.96–3.04)	0.07
Model 1	1.09 (0.67–1.78)	0.72	Ref	1.70 (0.994–3.06)	0.08
Model 2	1.10 (0.62–19.5)	0.74	Ref	1.63 (0.81–3.30)	0.17
2–5 years					
Crude model	1.02 (0.47–2.20)	0.95	Ref	0.85 (0.30–2.39)	0.75
Model 1	1.01 (0.45–2.22)	0.99	Ref	0.88 (0.31–2.51)	0.81
Model 2	1.15 (0.50–2.68)	0.74	Ref	1.07 (0.32–3.53)	0.91
5–19 year					
Crude model	**2.58 (1.43–4.67)**	**<0.01**	Ref	0.91 (0.44–1.87)	0.79
Model 1	**2.46 (1.35–4.47)**	**<0.01**	Ref	0.89 (0.43–1.85)	0.77
Model 2	**2.48 (1.30–4.76)**	**<0.01**	Ref	0.93 (0.42–2.09)	0.87

PICU, pediatric intensive care unit.

Model 1: adjusted for age and gender.

Model 2: additionally adjusted for Glasgow Coma Scale score status, fluctuations in serum sodium, and acute kidney injury.

Bold values represent the statistical significance at the *P*-value <0.05.

Table 3 reported the ORs and 95% CI of nutritional status for odds of PICU mortality in the total, as well as the sex and age-stratified subgroup analysis. In the total sample, underweight patients had higher odds of PICU mortality. The OR and 95% CI for this association were as follows: in the crude model (OR: 2.50; 95% CI: 1.37–4.53), adjusted model 1 (OR: 2.41; 95% CI: 1.32–4.41), and adjusted model 2 (OR: 2.12; 95% CI: 1.22–4.01). Gender-stratified analysis showed that the increased odds of PICU mortality for patients with underweight remained statistically significant in boys in the crude model (OR: 2.31; 95% CI: 1.19–4.50) and adjusted model 1 (OR: 2.29; 95% CI: 1.17–4.45). In model 2, there was no significant association between being underweight and PICU mortality among boys. Furthermore, no significant association among girls was found between underweight and PICU mortality, neither in crude nor adjusted models.

**Table 3:** The association between nutritional status measured by body mass *z*-score and risk of PICU mortality.

	Underweight (*Z*-score < −2)		Normal weight (−2 ≥ *Z*-score ≤ +1)	Overweight or obese (*Z*-score > +1)	
	OR (95% CI)	*P*-value		OR (95% CI)	*P*-value
Total sample					
Crude model	**2.50 (1.37–4.53)**	**<0.01**	Ref	1.04 (0.44–2.42)	0.93
Model 1	**2.41 (1.32–4.41)**	**<0.01**	Ref	1.20 (0.51–2.84)	0.67
Model 2	**2.12 (1.22–4.01)**	**0.02**	Ref	1.47 (0.57–3.75)	0.42
Gender stratification					
Boys					
Crude model	**2.31 (1.19–4.50)**	**0.01**	Ref	0.81 (0.28–2.28)	0.69
Model 1	**2.29 (1.17–4.45)**	**0.01**	Ref	0.98 (0.34–2.80)	0.97
Model 2	1.87 (0.90–3.85)	0.09	Ref	1.38 (0.41–4.64)	0.60
Girls					
Crude model	3.07 (0.75–12.47)	0.11	Ref	2.24 (0.44–11.22)	0.33
Model 1	3.05 (0.75–12.40)	0.12	Ref	2.22 (0.41–11.22)	0.33
Model 2	3.26 (.079–13.47)	0.10	Ref	2.14 (0.41–11.17)	0.37
Age stratification					
0–2 years					
Crude model	1.44 (0.66–3.14)	0.36	Ref	0.84 (0.27–2.66)	0.77
Model 1	1.40 (0.64–3.08)	0.40	Ref	1.01 (0.31–3.31)	0.98
Model 2	1.23 (0.52–2.91)	0.63	Ref	1.46 (0.34–6.26)	0.60
2–5 years					
Crude model	4.88 (1.00–23.68)	0.05	Ref	2.87 (0.39–21.26)	0.30
Model 1	4.05 (0.81–20.12)	0.09	Ref	2.99 (0.39–22.52)	0.29
Model 2	**9.67 (1.31–71.51)**	**0.03**	Ref	3.55 (0.34–36.98)	0.29
5–19 year					
Crude model	**5.41 (1.43–20.42)**	**0.01**	Ref	1.22 (0.20–7.46)	0.82
Model 1	**5.53 (1.44–21.16)**	**0.01**	Ref	1.24 (0.20–7.62)	0.81
Model 2	3.03 (0.73–12.59)	0.12	Ref	1.38 (0.21–8.95)	0.73

PICU, pediatric intensive care unit.

Model 1: adjusted for age and gender.

Model 2: additionally adjusted for Glasgow Coma Scale score status, fluctuations in serum sodium, and acute kidney injury.

Bold values represent the statistical significance at the *P*-value <0.05.

Age-stratified analysis showed that the increased odds of PICU mortality for underweight patients aged 0–2 years old were no longer statistically significant, neither in crude nor adjusted models. There was no significant association between being underweight and PICU mortality among patients aged 2–5 years old in the crude and adjusted model 1. Moreover, underweight patients aged 2–5 years old had higher odds of PICU mortality in the adjusted model 2 (OR: 9.67; 95% CI: 1.31–71.51). Furthermore, the age-stratified analysis demonstrated that underweight patients aged 5–19 years old had higher odds of PICU mortality in the crude model (OR: 5.41; 95% CI: 1.43–20.42) and adjusted model 1 (OR: 5.53; 95% CI: 1.44–21.16). However, in the adjusted model 2, no significant association between underweight and odds of PICU mortality was found among individuals aged 5–19 years old.

In addition, there was no significant association between being overweight or obese and odds of PICU mortality found in crude or adjusted models in total or in age- or gender-stratified analysis.

## Discussion

The present study sought to evaluate the association between malnutrition and odds of prolonged PICU stay and PICU mortality in Iranian critically ill children and adolescents. Our findings indicate that among critically ill children and adolescents, being underweight exhibits higher odds of experiencing a prolonged stay in the PICU and PICU mortality by 1.52 and 2.12 times, respectively. As well, the increased odds of prolonged PICU stay remained significant among underweight boys and individuals aged 5–19 years old. Furthermore, the heightened risk of PICU mortality was found to be significant among underweight children aged 2–5 years. In addition, there was no significant association between being overweight or obese and our clinical outcomes in total and age- or gender-stratified analysis.

The correlation between undernutrition and unfavorable outcomes can be attributed to diminished metabolic reserves, loss of muscle mass that affects respiratory function, and compromised immunity that results in delayed wound healing and heightened susceptibility to infection (1, 26, 27). It has been observed that malnourished patients in the PICU frequently receive inadequate nutrition, which can further deteriorate their prognosis (1). Our research shows a notable difference from the findings of two systematic reviews and meta-analyses (9, 28). These studies found no correlation between BMI status upon admission to the PICU and clinical outcomes (including duration of mechanical ventilation, hospital or PICU length of stay, and mortality) among critically ill children and adolescents. It should be noted that the included studies in these reviews were heterogeneous and lacked standardized nutritional assessment and classification methods. In line with our findings, according to the sensitivity analysis of the meta-analysis, which pooled studies used BMI-for-age *z*-scores as a standard nutritional classification method, underweight patients had significantly higher odds of PICU mortality by 35% (9). According to our findings, it seems that underweight patients experienced more severe illness status than normal-weight or overweight/obese ones. Interestingly, underweight patients exhibited nearly twice the baseline serum creatinine level in comparison to normal-weight or overweight/obese patients. Furthermore, we showed that the association between being underweight and higher odds of prolonged PICU stay and PICU mortality was independent of the GCS score status, serum sodium fluctuations, and AKI. In this context, further studies are required to evaluate the association between nutritional status and the risk of clinical outcomes in critically ill children and adolescents, taking into account the severity of their illness and other related risk factors.

The interpretation of BMI in children and adolescents depends on their age and gender (18). Therefore, we conducted subgroup analysis by age and gender to assess the impact of these factors on the association between nutritional status and PICU clinical outcome. Gender-specific subgroup analysis showed that the increased odds of prolonged PICU stay among underweight patients in the fully adjusted model remained significant among boys. It is important to note that 61% of boys and 39% of girls had a prolonged PICU stay. It seems that critically ill boys appeared to have a higher prevalence of moderate and severe GCS score status compared to girls [moderate GCS status: 59.4% of boys vs. 40.6% of girls; severe GCS status: 62.6% of boys vs. 34.7% of girls; (Supplementary Table S1)]. Consequently, this may explain why a higher number of critically ill boys compared to girls experienced a prolonged PICU stay. Moreover, the increased odds of PICU mortality after controlling for major confounders were insignificant, neither in boys nor girls. Furthermore, the prevalence of PICU mortality was 78.9% for boys and 21.1% for girls. It seems that additional risk factors, like severity of illness, which may not have been comprehensively considered, are associated with an increased risk of PICU mortality in both boys and girls. Furthermore, we found that underweight patients aged 5–19 years old had increased odds of prolonged PICU stay, while underweight patients aged 2–5 years old had increased odds of PICU mortality. To the best of our knowledge, it is important to note that no previous study has evaluated the association between nutritional status and outcomes among critically ill children and adolescents by conducting a subgroup analysis based on age and gender. It is unclear how gender and age contribute to the relationship between nutritional status and clinical outcomes in the PICU. It seems that there are biological differences in the response to critical illness or the response to interventions. This highlights the need for further investigation in order to understand better the impact of nutritional status on health outcomes while taking into account the age and gender of critically ill children and adolescents.

According to our results, we did not find any significant association between nutritional status and odds of prolonged PICU stay and PICU mortality among overweight/obese children and adolescents in total or age- or gender-stratified analysis. Our findings are consistent with previous meta-analyses (9, 13). Toh et al. showed that relative to normal-weight critically ill children and adolescents, overweight/obese counterparts did not have an increased risk of mortality (9). There were also no significant differences in the duration of mechanical ventilation, PICU, and hospital LOS between overweight/obese and normal-weight patients. Furthermore, our findings showed that overweight/obese patients, compared to underweight or normal-weight patients, may exhibit a better overall health status upon PICU admission. However, in trying to elucidate whether overweight/obese critically ill patients might have experienced poorer outcomes, numerous potential mechanisms have been proposed. It is believed that their chest wall compliance may be decreased, and upper airway obstruction may be increased (29, 30). This can potentially lead to ventilation issues (30). The estimation of medication dosages and fluid requirements based on the patient's weight also presents an obstacle that may affect the management of this particular patient group (11, 29, 31).

Conversely, it should be mentioned that improved outcomes are also observed in overweight/obese critically ill patients. This can be attributed to the obesity paradox, where the abundance of nutritional reserves and levels of adipokines play a significant role in providing protection (32–34). Our study did not find evidence to support either hypothesis, as we did not find a significant association between obesity and outcomes. This emphasizes the need for further research to comprehend better the relationship between overweight/obesity nutritional status and health outcomes in critically ill children and adolescents.

Early nutrition therapy is frequently advised for its holistic benefits in critically ill children (35, 36). However, it is important to note that it may inadvertently increase the risk of refeeding syndrome a critical condition, especially in undernourished patients (37). A gradual and cautious reintroduction of nutrition support is recommended to prevent and management of refeeding syndrome (37).

## Strength and limitation

Our study has revealed several strengths that contribute to the overall quality of our work. It is important to point out that not only participant descriptive characteristics confounders but also other risk factors like GCS score status, serum sodium fluctuations, and AKI upon the initial days of admission to the PICU were considered in our analysis. We also conducted a thorough analysis of age and gender subgroups in order to gain a better understanding of the potential association between nutritional status and risk of prolonged PICU stay and PICU mortality. However, it is important to note that some limitations should be taken into consideration. First, this study is limited by its retrospective nature, as it involves analyzing previously collected information rather than conducting a prospective randomized study. Second, we tried to evaluate the severity of illness status using descriptive and biochemical variables (including GCS score status, fluctuations in serum sodium, and AKI); however, it is recommended that the severity of illness be evaluated using validated severity of illness markers (such as the Pediatric index of mortality, the Pediatric risk of mortality, etc.). Third, we have no data regarding the participant's previous history of chronic disease, which may influence the severity of illness upon PICU admission. Third, despite controlling for major confounders in our analyses, the possibility of residual or unmeasured confounders cannot be ruled out.

## Conclusion

In conclusion, our findings indicate that critically ill children and adolescents who are underweight have higher odds of experiencing a prolonged stay in the PICU and PICU mortality compared to their normal-weight counterparts. These findings highlight the importance of careful monitoring upon admission for underweight pediatric patients in the PICU to improve their health outcomes.

## Data Availability

The raw data supporting the conclusions of this article will be made available by the authors, without undue reservation.
