# Supplementary material for: The association between nutritional status measured by body mass index and outcomes in the pediatric intensive care unit
Source: Front Pediatr. 2024 Sep 17;12:1421155. doi: 10.3389/fped.2024.1421155 (PMC11443694; doi:10.3389/fped.2024.1421155)
Supplement: Supplementary file 1 [file Table1.docx]

| **Supplementary table 1.** The characteristics of participants across gender. | | | | |
| --- | --- | --- | --- | --- |
|  | Total sample  (N=1015) | Boys  (N=574) | Girls  (N=441) | P-value^*^ |
| **Age (month)** | 54.3 ± 54.7 | 54.8 ± 5.7 | 53.6 ± 54.6 | 0.73 |
| **Height (cm)** | 97.6 ± 32.4 | 97.8 ± 33.3 | 97.2 ± 32.1 | 0.76 |
| **Weight (kg)** | 17.2 ± 14.8 | 17.3 ± 14.9 | 17.0 ± 14.6 | 0.76 |
| **Body mass index (kg/m^2^)** | 15.8 ± 7.3 | 15.8 ± 8.2 | 15.7 ± 5.8 | 0.87 |
| **Admission type** |  |  |  |  |
| Elective surgery (%) | 191 (18.8) | 112 (58.6) | 79 (41.4) | 0.48 |
| Emergency surgery (%) | 61 (6) | 38 (62.3) | 23 (37.7) |  |
| Medical (%) | 763 (75.2) | 424 (55.6) | 339 (44.4) |  |
| **Diagnosis category** |  |  |  |  |
| Cardiorespiratory (%) | 129 (12.7) | 76 (58.9) | 53 (41.1) | 0.7 |
| Neuromuscular (%) | 256 (25.2) | 140 (54.7) | 116 (45.3) |  |
| Infectious disease (%) | 77 (7.6) | 45 (58.4) | 32 (41.6) |  |
| Hematology/oncology (%) | 68 (6.7) | 34 (50) | 34 (50) |  |
| Gastrointestinal (%) | 129 (12.7) | 67 (51.9) | 62 (48.1) |  |
| Renal, endocrine (%) | 104 (10.2) | 62 (59.6) | 42 (40.4) |  |
| Surgical (%) | 191 (18.8) | 112 (58.6) | 79 (41.4) |  |
| Emergency (%) | 61 (6) | 38 (62.3) | 23 (37.7) |  |
| **Glasgow Coma Score Status** |  |  |  |  |
| Mild (%) | 668 (65.8) | 361 (54.0) | 307 (46.0) | 0.06 |
| Moderate (%) | 128 (12.6) | 76 (59.4) | 52 (40.6) |  |
| Severe (%) | 219 (21.6) | 137 (62.6) | 82(34.7) |  |
| **Serum sodium level (mmol/L)** | 136.9 ± 5.1 | 136.6 ± 4.9 | 136.8 ±5.3 | 0.39 |
| **Serum creatinine level (mg/dl)** | 0.66 ± 0.47 | 0.66 ± 0.50 | 0.65 ± 0.41 | 0.68 |
| **Acute kidney injury (%)** | 60 (5.9) | 32 (53.3) | 28 (46.7) | 0.62 |
| **Fluctuations in Serum Sodium levels** |  |  |  |  |
| Q1 | 561 (55.3) | 255 (45.4) | 306 (54.6) | 0.28 |
| Q2 | 174 (17.1) | 81 (46.5) | 93 (53.5) |  |
| Q3 | 94 (9.3) | 37 (39.3) | 57 (60.7) |  |
| Q4 | 186 (18.3) | 73 (42.2) | 113 (60.8) |  |
| **PICU length of stay (days)** | 5.9 ± 17.1 | 6.0 ± 17.7 | 5.9 ± 16.7 | 0.93 |
| **Prolonged PICU stay** (%) | 218 (21.5) | 133 (61.0) | 85 (39.0) | 0.13 |
| **PICU mortality** (%) | 57 (5.6) | 45 (78.9) | 12 (21.1) | <0.01 |
| Data represented as mean ± standard deviation, or median (interquartile) for continuous variables and number and percent for categorical variables.  *Chi-square or Fisher's exact, and analysis of variance or Kruskal-Wallis tests were used to test the categorical and continuous variables across nutritional status categories. | | | | |

| **Supplementary table 2.** The characteristics of participants across age groups. | | | | | |
| --- | --- | --- | --- | --- | --- |
|  | Total sample  (N=1015) | 0-2 years old  (N=464) | 2-5 years old  (N=178) | 5-19 years old  (N=373) | P-value^*^ |
| **Boys (%)** | 574 (56.6) |  |  |  |  |
| **Age (month)** | 54.3 ± 54.7 | 9.1 ± 6.6 | 41.7 ± 10.4 | 116.5 ± 39.3 | <0.01 |
| **Height (cm)** | 97.6 ± 32.4 | 69.7 ± 12.6 | 98.4 ± 16.1 | 131.8 ± 21.5 | <0.01 |
| **Weight (kg)** | 17.2 ± 14.8 | 7.7 ± 4.6 | 14.1 ± 7.9 | 30.4 ± 15.8 | <0.01 |
| **Body mass index (kg/m^2^)** | 15.8 ± 7.3 | 15.6 ± 8.6 | 14.5 ± 6.2 | 16.6 ± 5.5 | <0.01 |
| **Admission type** |  |  |  |  |  |
| Elective surgery (%) | 191 (18.8) | 113 (59.2) | 27 (14.1) | 51 (26.7) | <0.01 |
| Emergency surgery (%) | 61 (6) | 25 (41.0) | 14 (23.0) | 22 (36.1) |  |
| Medical (%) | 763 (75.2) | 326 (42.7) | 137 (18.0) | 300 (39.3) |  |
| **Diagnosis category** |  |  |  |  |  |
| Cardiorespiratory (%) | 129 (12.7) | 74 (57.4) | 21 (16.3) | 34 (26.4) | <0.01 |
| Neuromuscular (%) | 256 (25.2) | 95 (37.1) | 42 (16.4) | 119 (46.5) |  |
| Infectious disease (%) | 77 (7.6) | 25 (32.5) | 15 (19.5) | 37 (48.1) |  |
| Hematology/oncology (%) | 68 (6.7) | 14 (20.6) | 14 (20.6) | 40 (58.8) |  |
| Gastrointestinal (%) | 129 (12.7) | 72 (55.8) | 24 (18.6) | 33 (25.6) |  |
| Renal, endocrine (%) | 104 (10.2) | 46 (44.2) | 21 (20.2) | 37 (35.6) |  |
| Surgical (%) | 191 (18.8) | 113 (59.2) | 27 (14.1) | 51 (26.7) |  |
| Emergency (%) | 61 (6) | 25 (41.0) | 14 (23.0) | 22 (36.1) |  |
| **Severity of illness** |  |  |  |  |  |
| Mild (%) | 668 (65.8) | 302 (45.2) | 109 (16.3) | 257 (38.5) | 0.34 |
| Moderate (%) | 128 (12.6) | 59 (46.1) | 29 (22.7) | 40 (31.3) |  |
| Severe (%) | 219 (21.6) | 103 (47.0) | 40 (18.3) | 76 (34.7) |  |
| **Serum sodium level (mmol/L)** | 136.9 ± 5.1 | 136.9 ± 5.5 | 136.5 ± 3.9 | 136.7 ± 5.1 | 0.28 |
| **Serum creatinine level (mg/dl)** | 0.66 ± 0.47 | 0.58 ± 0.34 | 0.64 ± 0.75 | 0.74 ± 0.62 | <0.01 |
| **Acute kidney injury (%)** | 60 (5.9) | 28 (46.6) | 7 (11.7) | 25 (41.7) | 0.46 |
| **Fluctuations in Serum Sodium levels** |  |  |  |  |  |
| Q1 | 561 (55.3) | 256 (45.6) | 101 (18.0) | 209 (36.4) | 0.68 |
| Q2 | 174 (17.1) | 73 (41.9) | 29 (16.6) | 75 (41.5) |  |
| Q3 | 94 (9.3) | 47 (50.0) | 18 (19.1) | 35 (30.9) |  |
| Q4 | 186 (18.3) | 88 (47.3) | 30 (16.1) | 54 (36.6) |  |
| **PICU length of stay (days)** | 5.9 ± 17.1 | 6.5 ± 19.4 | 5.3 ± 17.2 | 5.6 ± 17.5 | 0.65 |
| **Prolonged PICU stay** (%) | 218 (21.5) | 107 (49.1) | 40 (18.3) | 71 (32.6) | 0.35 |
| **PICU mortality** (%) | 57 (5.6) | 31 (54.4) | 12 (21.1) | 14 (24.6) | 0.17 |
| Data represented as mean ± standard deviation, or median (interquartile) for continuous variables and number and percent for categorical variables.  *Chi-square or Fisher's exact, and analysis of variance or Kruskal-Wallis tests were used to test the categorical and continuous variables across nutritional status categories. | | | | | |
